# Supplementary figures and images for: Primary care-based screening and management of depression amongst heavy drinking patients: Interim secondary outcomes of a three-country quasi-experimental study in Latin America
Source: PLoS One. 2021 Aug 5;16(8):e0255594. doi: 10.1371/journal.pone.0255594 (PMC8341512; doi:10.1371/journal.pone.0255594)

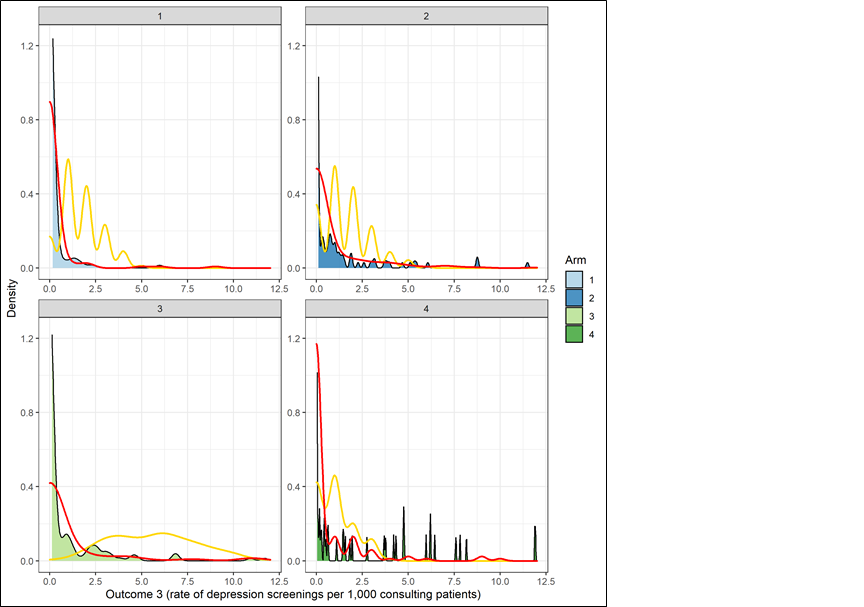

Supplement: S1 Fig — The red and yellow lines indicate hypothetical negative binomial and poisson distributions, respectively, generated from parameters of the empirical arm-specific distribution. (TIF) [file pone.0255594.s001.tif]
