# Supplementary material for: Primary care-based screening and management of depression amongst heavy drinking patients: Interim secondary outcomes of a three-country quasi-experimental study in Latin America
Source: PLoS One. 2021 Aug 5;16(8):e0255594. doi: 10.1371/journal.pone.0255594 (PMC8341512; doi:10.1371/journal.pone.0255594)
Supplement: S1 Table — (DOCX) [file pone.0255594.s002.docx]

| **S1 Table. Results of regression analyses for evaluating Hypotheses 1-3 for outcome 1 (cumulative share of heavy drinking patients assessed for depression)** | | | |
| --- | --- | --- | --- |
|  | Hypothesis 1 | Hypothesis 2 | Hypothesis 3 |
| Exposure ^a^ | 1.75 (0.58 to 5.64; 0.328) | 3.04 (0.12 to 76.86; 0.464) | 0.36 (0.11 to 1.04; 0.071) |
| Country (base: Colombia) |  |  |  |
| Mexico | 10.88 (3.04 to 52.04; 0.002) | 2.33 (0.23 to 39.08; 0.500) | 1.80 (0.60 to 5.21; 0.284) |
| Peru | 27.12 (2.77 to 1174.98; 0.019) | 7.00 (0.18 to 10647.32; 0.382) | 23.74 (1.66 to 6754.61; 0.069) |
| Female (base: male) | 1.04 (0.2 to 3.56; 0.954) | 1.24 (0.13 to 10.56; 0.843) | 1.28 (0.38 to 4.06; 0.681) |
| Age | 1.03 (0.97 to 1.12; 0.409) | 1.17 (0.99 to 1.50; 0.137) | 1.05 (0.99 to 1.13; 0.203) |
| Doctor (base: other profession) | 0.83 (0.26 to 2.61; 0.749) | 0.80 (0.09 to 7.06; 0.832) | 1.34 (0.40 to 4.34; 0.626) |
| Intercept | 0.90 (0.08 to 9.87; 0.928) | 0.01 (0.00 to 2.34; 0.149) | 1.15 (0.06 to 16.51; 0.921) |
| Observations | 82 | 55 | 73 |
| Note. Presented are exponentiated coefficients of fractional response regression analyses, which should be interpreted as percentage increase associated with one unit increase in predictor variable.  Numbers in brackets denote: 95% confidence intervals; p-value  ^a^ Exposure variable defined by hypothesis: H1: without (base) vs with municipal support, H2: without (base) vs with training, H3: short (base) vs standard package | | | |
