# Supplementary material for: Primary care-based screening and management of depression amongst heavy drinking patients: Interim secondary outcomes of a three-country quasi-experimental study in Latin America
Source: PLoS One. 2021 Aug 5;16(8):e0255594. doi: 10.1371/journal.pone.0255594 (PMC8341512; doi:10.1371/journal.pone.0255594)
Supplement: S2 Table — (DOCX) [file pone.0255594.s003.docx]

| **S2 Table. Results of fractional response regression analyses for evaluating Hypotheses 1-3 for outcome 2 (cumulative share of patients at-risk for depression receiving appropriate interventions)** | | | |
| --- | --- | --- | --- |
|  | Hypothesis 1 | Hypothesis 2 | Hypothesis 3 |
| Exposure ^a^ | 0.25 (0.06 to 0.95; 0.055) | 1.15 (0.07 to 14.89; 0.912) | 0.83 (0.21 to 3.19; 0.784) |
| Country (base: Colombia) |  |  |  |
| Mexico | 0.07 (0.00 to 0.67; 0.038) | ^b^ | 0.05 (0.00 to 0.40; 0.012) |
| Peru | 1.53 (0.06 to 26.39; 0.771) | ^b^ | 14.51 (0.68 to 671.56; 0.120) |
| Female (base: male) | 0.27 (0.04 to 1.45; 0.154) | 0.66 (0.07 to 5.21; 0.707) | 0.31 (0.06 to 1.36; 0.140) |
| Age | 0.98 (0.92 to 1.04; 0.473) | 1.06 (0.99 to 1.16; 0.138) | 0.90 (0.81 to 0.98; 0.042) |
| Doctor (base: other profession) | 0.27 (0.04 to 1.72; 0.184) | 0.03 (0.00 to 0.21; 0.006) | 0.19 (0.03 to 1.10; 0.070) |
| Intercept | 117.94 (2.94 to 27863.66; 0.038) | 1.69 (0.01 to 240.78; 0.828) | 504.35 (5.65 to 139591.35; 0.018) |
| Observations | 53 | 34 | 51 |
| Note. Presented are exponentiated coefficients of fractional response regression analyses, which should be interpreted as percentage increase associated with one unit increase in predictor variable.  Numbers in brackets denote: 95% confidence intervals; p-value  ^a^ Exposure variable defined by hypothesis: H1: without (base) vs with municipal support, H2: without (base) vs with training, H3: short (base) vs standard package  ^b^ Inclusion of country variables would have inflated the standard errors and were therefore excluded from the models. | | | |
