# Supplementary material for: Primary care-based screening and management of depression amongst heavy drinking patients: Interim secondary outcomes of a three-country quasi-experimental study in Latin America
Source: PLoS One. 2021 Aug 5;16(8):e0255594. doi: 10.1371/journal.pone.0255594 (PMC8341512; doi:10.1371/journal.pone.0255594)
Supplement: S3 Table — (DOCX) [file pone.0255594.s004.docx]

| **S3 Table. Results of negative binomial regression analyses for evaluating Hypotheses 1-3 for outcome 3 (cumulative rate of depression screens per 1,000 consulting patients)** | | | |
| --- | --- | --- | --- |
|  | Hypothesis 1 | Hypothesis 2 | Hypothesis 3 |
| Exposure ^a^ | 0.90 (0.63 to 1.29; 0.572) | 3.52 (1.70 to 7.82; 0.002) | 1.27 (0.89 to 1.80; 0.185) |
| Country (base: Colombia) |  |  |  |
| Mexico | 2.93 (1.97 to 4.38; 0.00000) | 2.67 (1.21 to 6.10; 0.015) | 2.86 (2.00 to 4.10; 0.00000) |
| Peru | 1.24 (0.73 to 2.08; 0.432) | 1.03 (0.49 to 2.17; 0.945) | 1.88 (1.06 to 3.33; 0.031) |
| Female (base: male) | 0.51 (0.32 to 0.79; 0.003) | 0.51 (0.27 to 0.98; 0.047) | 0.76 (0.50 to 1.16; 0.200) |
| Age | 1.00 (0.99 to 1.02; 0.547) | 1.03 (1.01 to 1.06; 0.011) | 0.99 (0.98 to 1.01; 0.385) |
| Doctor (base: other profession) | 0.39 (0.26 to 0.59; 0.00001) | 0.42 (0.19 to 0.85; 0.018) | 0.55 (0.36 to 0.84; 0.005) |
| Intercept | 1.06 (0.46 to 2.48; 0.884) | 0.12 (0.03 to 0.42; 0.001) | 0.85 (0.31 to 2.29; 0.736) |
| Observations | 349 | 309 | 287 |
| Log Likelihood | -390.95 | -181.58 | -410.37 |
| theta | 1.08 (0.19) | 0.91^***^ (0.23) | 1.38^***^ (0.26) |
| Akaike Inf. Crit. | 795.91 | 377.16 | 834.73 |
| Note. Presented are exponentiated coefficients of negative binomial regression analyses, which should be interpreted as Incidence Rate Ratios.  Numbers in brackets denote: 95% confidence intervals; p-value  ^a^ Exposure variable defined by hypothesis: H1: without (base) vs with municipal support, H2: without (base) vs with training, H3: short (base) vs standard package  ^b^ Inclusion of country variables would have inflated the standard errors and were therefore excluded from the models. | | | |
