# Supplementary material for: Primary care-based screening and management of depression amongst heavy drinking patients: Interim secondary outcomes of a three-country quasi-experimental study in Latin America
Source: PLoS One. 2021 Aug 5;16(8):e0255594. doi: 10.1371/journal.pone.0255594 (PMC8341512; doi:10.1371/journal.pone.0255594)
Supplement: S4 Table — (DOCX) [file pone.0255594.s005.docx]

| **S4 Table. Results from additional analyses testing the hypotheses with inclusion of interaction effects of country and exposure variables (only reported for Outcome 3 - Hypothesis 2).** | |
| --- | --- |
| Training (base: no training) | 71.82 (5.84 to 280126.55; 0.031) |
| Country (base: Colombia) |  |
| Mexico | 58.18 (3.46 to 235896.72; 0.048) |
| Peru | 38.68 (2.41 to 155908.44; 0.073) |
| Interaction of Exposure^a^ and |  |
| Mexico | 0.04 (0.00 to 0.64; 0.107) |
| Peru | 0.02 (0.00 to 0.31; 0.051) |
| Female (base: male) | 0.47 (0.24 to 0.91; 0.029) |
| Age | 1.03 (1.01 to 1.06; 0.014) |
| Doctor (base: other profession) | 0.40 (0.19 to 0.83; 0.015) |
| Intercept | 0.01 (0.00 to 0.12; 0.018) |
| Observations | 309 |
| Log Likelihood | -176.81 |
| theta | 0.95 (0.24) |
| Akaike Inf. Crit. | 371.63 |
| Note. Presented are exponentiated coefficients of fractional response regression analyses, which should be interpreted as percentage increase associated with one unit increase in predictor variable. Outcome variable: cumulative rate of depression screens per 1,000 consulting patients  Numbers in brackets denote: 95% confidence intervals; p-value  ^a^ Exposure variable defined by hypothesis: H1: without (base) vs with municipal support, H2: without (base) vs with training, H3: short (base) vs standard package | |
