# Supplementary material for: Primary care-based screening and management of depression amongst heavy drinking patients: Interim secondary outcomes of a three-country quasi-experimental study in Latin America
Source: PLoS One. 2021 Aug 5;16(8):e0255594. doi: 10.1371/journal.pone.0255594 (PMC8341512; doi:10.1371/journal.pone.0255594)
Supplement: S5 Table — (DOCX) [file pone.0255594.s006.docx]

| **S5 Table. Results from sensitivity analyses testing the effect of engaging in training (per-protocol analyses)** | | | | | |
| --- | --- | --- | --- | --- | --- |
|  | Outcome 1^a^ | Outcome 2 ^a^ | Outcome 3 ^a^ | Outcome 3 ^a^ –  only Arm 2 |  |
| Training (base: no training) | 5.00 (0.60 to 75.21; 0.173) | 0.55 (0.05 to 4.75; 0.609) | 3.69 (2.10 to 6.68; 0.00001) | 2.69 (1.28 to 5.90; 0.008) |  |
| Country (base: Colombia) |  |  |  |  |  |
| Mexico | 3.48 (0.36 to 67.00; 0.333) | ^b^ | 3.64 (1.71 to 8.15; 0.001) | 2.86 (1.21 to 7.17; 0.013) |  |
| Peru | 10.44 (0.31 to 6488.73; 0.270) | ^b^ | 1.31 (0.63 to 2.76; 0.472) | 0.80 (0.36 to 1.76; 0.573) |  |
| Female (base: male) | 1.92 (0.27 to 13.97; 0.506) | 0.52 (0.05 to 4.38; 0.568) | 0.72 (0.37 to 1.38; 0.323) | 0.50 (0.24 to 1.01; 0.050) |  |
| Age | 1.16 (1.00 to 1.46; 0.127) | 1.06 (0.99 to 1.16; 0.109) | 1.02 (1.00 to 1.04; 0.113) | 1.02 (1.00 to 1.05; 0.082) |  |
| Doctor (base: other profession) | 1.40 (0.20 to 10.59; 0.736) | 0.02 (0.00 to 0.18; 0.005) | 0.58 (0.27 to 1.18; 0.127) | 0.45 (0.19 to 1.00; 0.042) |  |
| Intercept | 0.01 (0.00 to 0.69; 0.067) | 3.62 (0.05 to 387.76; 0.559) | 0.13 (0.04 to 0.39; 0.0004) | 0.28 (0.07 to 1.02; 0.048) |  |
| Observations | 55 | 34 | 309 | 192 |  |
| Log Likelihood |  |  | -177.26 | -150.77 |  |
| theta |  |  | 1.01 (0.26) | 1.09^***^ (0.30) |  |
| Akaike Inf. Crit. |  |  | 368.53 | 315.54 |  |
| Note. For outcome 1 and 2, exponentiated coefficients of fractional response regression analyses are presented, which should be interpreted as percentage increase associated with one unit increase in predictor variable. For outcome 3, exponentiated coefficients of negative binomial regression analyses, which should be interpreted as Incidence Rate Ratios.  Numbers in brackets denote: 95% confidence intervals; p-value  ^a^ Outcome 1: cumulative share of heavy drinking patients assessed for depression; Outcome 2: cumulative share of patients at-risk for depression receiving appropriate interventions; Outcome 3: cumulative rate of depression screens per 1,000 consulting patients  ^b^ Inclusion of country variables would have inflated the standard errors and were therefore excluded from the models. | | | | | |
