# Supplementary material for: Primary care-based screening and management of depression amongst heavy drinking patients: Interim secondary outcomes of a three-country quasi-experimental study in Latin America
Source: PLoS One. 2021 Aug 5;16(8):e0255594. doi: 10.1371/journal.pone.0255594 (PMC8341512; doi:10.1371/journal.pone.0255594)
Supplement: Attachment — Submitted filename: Response to reviewers comments.docx [file pone.0255594.s007.docx]

**PONE-D-21-06289 - Primary care-based screening and management of depression amongst heavy drinking patients: Secondary outcomes of a three-country quasi-experimental study in Latin America**

| **Comment** | **Response** |
| --- | --- |
| **Journal Requirements:** | |
| Please ensure that your manuscript meets PLOS ONE's style requirements, including those for file naming. The PLOS ONE style templates can be found at https://journals.plos.org/plosone/s/file?id=wjVg/PLOSOne_formatting_sample_main_body.pdf and https://journals.plos.org/plosone/s/file?id=ba62/PLOSOne_formatting_sample_title_authors_affiliations.pdf | We have reviewed and edited our manuscript in line with PLOS ONE’s style requirements. |
| Please include a separate caption for each figure in your manuscript. | We have added captions for Figs 1 and 2. |
| Please review your reference list to ensure that it is complete and correct. If you have cited papers that have been retracted, please include the rationale for doing so in the manuscript text or remove these references and replace them with relevant current references. Any changes to the reference list should be mentioned in the rebuttal letter that accompanies your revised manuscript. If you need to cite a retracted article, indicate the article’s retracted status in the References list and also include a citation and full reference for the retraction notice. | Additional references have been added as part of our response to reviewers’ feedback.  We have edited and corrected all references (removing the duplicate reference Gilbody et al 2003). |
| **Reviewer #1:** | |
| Title must be modify since authors presented partial, preliminary results because restrictions due to COVID 19. It was not possible to determine the community support on depression activity rates. In the same line, was not completed the evaluation on clinical intervention package (less versus more intensive) for screening and advice delivery by PHC. | We have revised the title accordingly to “Primary care-based screening and management of depression amongst heavy drinking patients: Interim secondary outcomes of a three-country quasi-experimental study in Latin America”, See p1 line 3. |
| Introduction must inform of similar studies and their findings in LIC and HIC so it is possible to compare and discuss afterwards. | We have added additional content to the introduction outlining evidence for the use of collaborative care models in both contexts, and of increasing positive evidence for psychological interventions for co-morbid AUD and depression. See p6 line 128 to p7 line 130. |
| The design story must tell that this survey is part of the international SCALA project to better understand the characteristics of municipal areas, even more if they were not randomly selected. | We have added more specific detail on which districts comprise the intervention versus municipal areas in each country. See p8 lines 165-171. |
| It would be important to detail providers profession and their training in depression since PHQ-2 don't evaluate suicide risk. | We have highlighted that profession varied across providers in the methods section (see p8 lines 174-175) and added numbers of participating providers per professional group to the Sample characteristics section of our Results (see p17 p348-350). We do not have detailed information on their previous training in alcohol/depression prevention or management so have added this as a limitation (see p26 lines 524-529). |
| In this point it would be interesting to clarify what happened with 83 persons that didn't receive treatment (from 615 heavy drinking persons, 230 qualify for depression too. From these 230, 147 (63,9%) received treatment, so were left 83 persons without treatment?) | We do not currently have additional data to explain the shortfall in care provision. We hope to investigate this issue further as part of the forthcoming process evaluation interviews with providers. We have added content to the manuscript to acknowledge this issue in our Discussion, see p26 lines 524-525. |
| In the discuss section, would be interesting to contrast heavy drinking + depression findings 889.4%) with other results in the literature. | Just to clarify, the 89.4% represents the screening rate of those patients identified as heavy drinkers – it does not necessarily mean that they did have depression.  We are not aware of other relevant literature on whether this represents a high or low rate of screening. However, if the reviewer could possibly advise further, we would be happy to revisit this point. |
| **Reviewer #2:** | |
| This is a very nicely written paper describing implementation of alcohol and depression screening in primary care in 3 Latin American countries. This topic is of high importance given the generally low rates of uptake of alcohol and depression screening in routine practice. The authors also offer compelling next steps and future directions. I would suggest adding a bit more detail in a few places as outlined in my specific comments below. | Thank you for your positive and constructive feedback – we have endeavoured to respond to your specific comments as below. |
| 1) There is a large body of literature on collaborative care for depression as well as implementation of alcohol screening and intervention. It would be important to situate this study more in that literature in both the introduction and discussion sections. | We have added additional content to the introduction outlining evidence for the use of collaborative care models in both contexts, See p6 line 128 to p7 line 130. We have also added reflections on this point to the discussion, see p23, lines 454-457. |
| 2) The field of implementation science offers multiple implementation frameworks as well as discrete implementation strategies to improve uptake of health services. How does this study connect to that broader literature? Do your intervention strategies at the municipal and clinical levels draw from this literature? | In terms of specific frameworks, we used the Tailored Implementation in Chronic Disease (TICD) checklist to tailor clinical materials for local use (see p9 line 193) and the Institute for Healthcare Improvement Going to Scale Framework (see p10 line 209) to inform the development of the community action and support measures. Selection of specific strategies/measures was based on existing empirical evidence of ‘what works’ to promote AUD and depression care implementation in PHC settings. |
| 3) The hypotheses are not presented until far into the method section. It would help the reader to have those presented earlier, such as at the end of the introduction section. | We have moved the hypotheses to the end of the introduction as suggested, see p7 lines 138-144. |
| 4) On page 9, it would be helpful for the authors to elaborate more on this sentence “due to the COVID-19 pandemic, only limited implementation of these measures was possible”. This would be a major factor in testing hypotheses 1 and 3. While there is mention of it in the limitations section, it would help the reader to better understand this early on in order to fully assess the results. | We have added a statement to highlight the particular impact on plans for municipal communication activities to p11, lines 219-220. |
| 5) Booster sessions were mentioned in the intervention package section (pages 8-9). What did these entail? | We have added a brief description of booster session content to p10, lines 202-204. |
